# Abscisic acid mediated proline biosynthesis and antioxidant ability in roots of two different rice genotypes under hypoxic stress

**DOI:** 10.1186/s12870-020-02414-3

**Published:** 2020-05-08

**Authors:** Xiaochuang Cao, Longlong Wu, Meiyan Wu, Chunquan Zhu, Qianyu Jin, Junhua Zhang

**Affiliations:** 1 State Key Laboratory of Rice Biology, China National Rice Research Institute, No. 359 Tiyuchang Road, Hangzhou, 310006 People’s Republic of China; 2 Hubei Collaborative Innovation Center for Grain Industry/Engineering Research Center of Ecology and Agricultural Use of Wetland, Ministry of Education, Yangtze University, Jingzhou, 434025 Hubei China

**Keywords:** Abscisic acid, Proline metabolism, Root oxidative damage, Hypoxic stress, Rice

## Abstract

**Background:**

Abscisic acid (ABA) and proline play important roles in rice acclimation to different stress conditions. To study whether cross-talk exists between ABA and proline, their roles in rice acclimation to hypoxia, rice growth, root oxidative damage and endogenous ABA and proline accumulation were investigated in two different rice genotypes (‘Nipponbare’ (Nip) and ‘Upland 502’ (U502)).

**Results:**

Compared with U502 seedlings, Nip seedlings were highly tolerant to hypoxic stress, with increased plant biomass and leaf photosynthesis and decreased root oxidative damage. Hypoxia significantly stimulated the accumulation of proline and ABA in the roots of both cultivars, with a higher ABA level observed in Nip than in U502, whereas the proline levels showed no significant difference in the two cultivars. The time course variation showed that the root ABA and proline contents under hypoxia increased 1.5- and 1.2-fold in Nip, and 2.2- and 0.7-fold in U502, respectively, within the 1 d of hypoxic stress, but peak ABA production (1 d) occurred before proline accumulation (5 d) in both cultivars. Treatment with an ABA synthesis inhibitor (norflurazon, Norf) inhibited proline synthesis and simultaneously aggravated hypoxia-induced oxidative damage in the roots of both cultivars, but these effects were reversed by exogenous ABA application. Hypoxia plus Norf treatment also induced an increase in glutamate (the main precursor of proline). This indicates that proline accumulation is regulated by ABA-dependent signals under hypoxic stress. Moreover, genes involved in proline metabolism were differentially expressed between the two genotypes, with expression mediated by ABA under hypoxic stress. In Nip, hypoxia-induced proline accumulation in roots was attributed to the upregulation of Os*P5CS2* and downregulation of Os*ProDH*, whereas upregulation of Os*P5CS1* combined with downregulation of Os*ProDH* enhanced the proline level in U502.

**Conclusion:**

These results suggest that the high tolerance of the Nip cultivar is related to the high ABA level and ABA-mediated antioxidant capacity in roots. ABA acts upstream of proline accumulation by regulating the expression of genes encoding the key enzymes in proline biosynthesis, which also partly improves rice acclimation to hypoxic stress. However, other signaling pathways enhancing tolerance to hypoxia in the Nip cultivar still need to be elucidated.

## Background

As the aerobic organisms, higher plants require oxygen (O_2_) to support respiration, metabolism and growth. However, plants often experience the hypoxic stress due to the low O_2_ concentration, that induced by the long-time flooding, waterlogging, or soil compaction [1, 2]. Depletion of O_2_, the terminal electron acceptor in the mitochondrial respiratory chain, induces a significant decrease in ATP synthesis and causes the excessive accumulation of reactive oxygen species (ROS) [3, 4]. ROS are cytotoxic and disrupt normal cell metabolism by oxidatively damaging lipids and proteins, thus causing pigment breakdown, leakage of cellular contents and eventually cell death [5, 6]. Low O_2_ also affects plant nutrient metabolism and growth, as indicated by the significant alterations in root morphology, nutrient uptake and expression of genes associated with these processes [7–9].

To cope with hypoxic stress, plants have developed several mechanisms to reduce the negative effects of hypoxia [9]. Morphological adaptations, including the formation of adventitious roots and aerenchyma, and the alterations of leaf thickness, serve as escape strategies utilized by plants [10, 11]. Cell wall lignification and suberization in root cortices and steles also prevent O_2_ loss from the roots during hypoxia [12, 13]. Compared to hypoxia-sensitive plants, hypoxia-tolerant plants develop the more adventitious root numbers and higher radial oxygen loss to avoid O_2_ deficiency [13, 14]. At the cellular and physiological levels, hypoxia-tolerant plants usually evolve a number of antioxidative enzymes to scavenge ROS (such as hydrogen peroxidase, CAT; ascorbate peroxidase, APX; peroxidase, POD; and superoxide dismutase, SOD) and several complex metabolic adaptations [4, 15]. Mechanisms for the protection of proline, a compatible osmolyte, have been proposed and are particularly associated with adaptation to hypoxic [16], osmotic [17], salinity [18], heavy metal [19], and freezing [20] stresses. A number of possible functions have been proposed, including enabling osmotic adjustment, stabilizing protein and cell membrane structures, and acting as free radical scavengers [21–23]. Other proposed functions of proline include regulation of cytosolic acidity, transfer of energy and reductant activity, acting as a carbon and nitrogen reserve and as a signaling molecule [24, 25].

Plant hormones, especially abscisic acid (ABA), also play important roles in eliciting chemical responses governing metabolism involved in plant responses to a wide range of abiotic stresses [26]. Under hypoxic condition, an increase in ABA content has been reported in the roots of different species, and application of exogenous ABA significantly increased anoxia tolerance in Arabidopsis [27]. These adaptive responses to abiotic stress induce distinct physiological and biochemical changes, such as activation of stomatal closure, antioxidative enzymes, adventitious root formation, and carbohydrates of metabolites [28, 29]. Some authors further demonstrated that the functions of proline and the regulation of proline metabolism are dependent on ABA accumulation [30, 31], whereas other responses occur independently of ABA, and that ABA alone cannot duplicate drought-induced proline accumulation [32]. The responses of ABA accumulation to abiotic stress significantly vary with the plant species, varieties and organs [27, 33]. Therefore, understanding these protective mechanisms will contribute to the genetic modification of plants for improving adaptation to harsh environmental conditions.

Both lowland rice and upland rice are important rice cultivars in northern China. The roots of these two rice genotypes display distinctive morphological, hormone-related and gene expression features, and these differences play important roles in detoxification, defense against oxidative stress, maintenance of cell turgor and integrity, and protein synthesis [34, 35]. In our study, the differences in the accumulation of endogenous ABA and proline, root oxidative damage, and their protective mechanisms in rice acclimation to hypoxic stress were investigated using hydroponic cultivation. Two different rice cultivars, namely, the lowland rice cultivar ‘Nipponbare’ (Nip) and the upland rice cultivar ‘Upland 502’ (U502), were selected and cultivated under hypoxic and normoxic conditions. Our results demonstrate that ABA-mediated proline accumulation and antioxidant ability in roots likely plays an important role in enhancing adaptation to hypoxic stress, especially in lowland cultivar. However, other ABA-mediated signal regulation mechanisms involved in rice tolerance to hypoxia still need further investigation.

## Results

### Rice growth and physiological characteristics

**Fig. 1:**
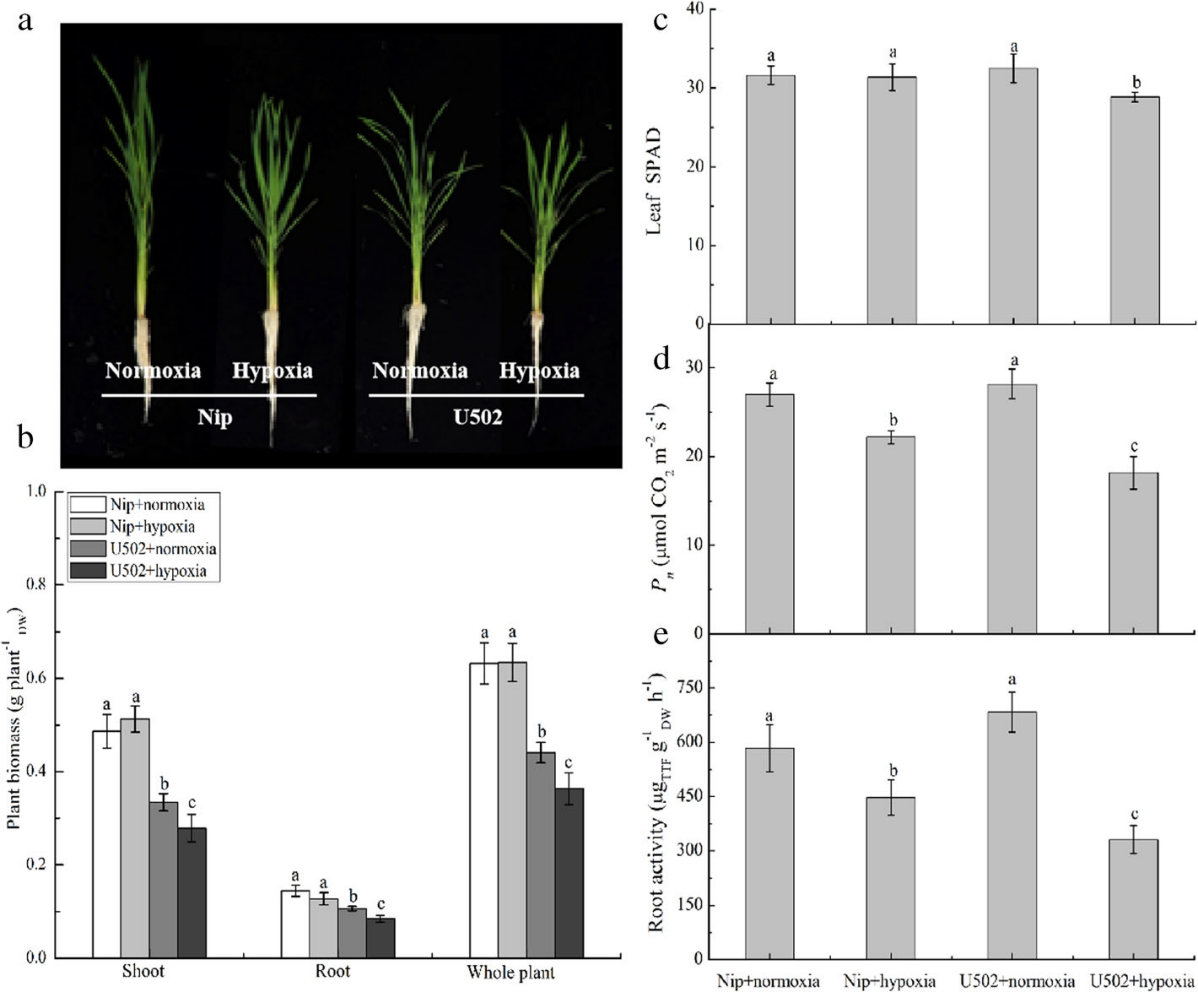
Growth traits (**a**), biomass (**b**), leaf SPAD values (**c**), photosynthesis (*P*_*n*_, **d**) and root activity (**e**) of both rice cultivars in response to normoxic and hypoxic conditions after 14 d of cultivation. Nip and U502 represent the lowland japonica rice ‘Nipponbare’ and the upland japonica rice ‘Upland 502’, respectively (here and below). DW represents dry weight. Data are the mean ± SE of three independent experiments. Different letters indicate significant differences at *P* < 0.05 using the least significant difference (LSD) test

Growth and physiology-related parameters such as biomass, photosynthesis rate and root activity in both genotypes were influenced differently in response to hypoxic stress (Fig. 1a-e). Compared with the normoxia treatment, hypoxia significantly reduced rice biomass (e.g., root, shoot and whole-plant biomass) and leaf SPAD values in the U502 cultivar, whereas these parameters in the Nip cultivar displayed no significant changes. Both leaf photosynthesis and root activity were significantly suppressed under hypoxia, with 17.9 and 23.3% decreases, respectively, in the Nip cultivar and 34.8 and 51.6% decreases, respectively, in the U502 cultivar, and their values in Nip cultivar were significantly higher than those in U502 cultivar (Fig. 1d, e).

To determine the level of oxidative damage in roots under hypoxic stress, the levels of both lipid peroxidation (MDA) and protein oxidation (carbonyl groups), and the activities of antioxidant enzymes were further investigated. In contrast with the effects on growth, hypoxia enhanced the root oxidative damage in U502, as evidenced by the significantly higher MDA and carbonyl contents in the U502 than in the Nip cultivar (Table 1). However, hypoxia showed no significant effect on root oxidative damage in the Nip cultivar. Correspondingly, the activities of SOD, POD, CAT, and APX in roots significantly increased in the U502, but only POD and APX activities increased in the Nip, relative to the activities under normoxic condition. Therefore, the Nip cultivar is seemingly more tolerant to hypoxic stress than the U502 cultivar.

**Table 1:** The levels of lipid peroxidation (MDA) and protein oxidation (Carbonyl), and activities of antioxidant enzymes in roots of rice plants subjected to hypoxia and normoxia conditions. Data represent means±SE (*n* = 3). Different letters indicate significant differences at *P* < 0.05 using the least significant difference (LSD) test. DW, dry weight; Nip, Nipponbare, Oryza. Sativa L. spp. japonica; U502, Upland rice 502

Enzymes^a^	Nip		U502	
	Hypoxia	Normoxia	Hypoxia	Normoxia
MDA (nmol g^−1^_DW_)	24.2 ± 1.1b	24.2 ± 1.6b	34.2 ± 0.2a	24.7 ± 2.1b
Carbonyl (μmol g^−1^ Protein)	54.7 ± 3.2b	50.5 ± 2.6bc	67.4 ± 1.6a	46.2 ± 2.1c
SOD (U g^−1^_DW_)	284.7 ± 22.6c	317.9 ± 21.1c	782.6 ± 67.4a	398.4 ± 19.5b
POD (U g^−1^_DW_)	35,105 ± 4263a	16,911 ± 3674b	38,153 ± 2063a	19,379 ± 2658b
CAT (mmol min^−1^ g^− 1^_DW_)	426.3 ± 33.2a	419.5 ± 28.9a	375.3 ± 28.4a	285.8 ± 23.2b
APX (μmol min^−1^ g^− 1^_DW_)	1.3 ± 0.1a	0.7 ± 0.1b	1.2 ± 0.1a	0.8 ± 0.1b

^a^*MDA* Malondialdehyde, *SOD* Superoxide Dismutase, *POD* Peroxidase, *CAT* Catalase, *APX* Aseorbateperoxidase

### Rice endogenous proline and ABA accumulation

**Fig. 2:**
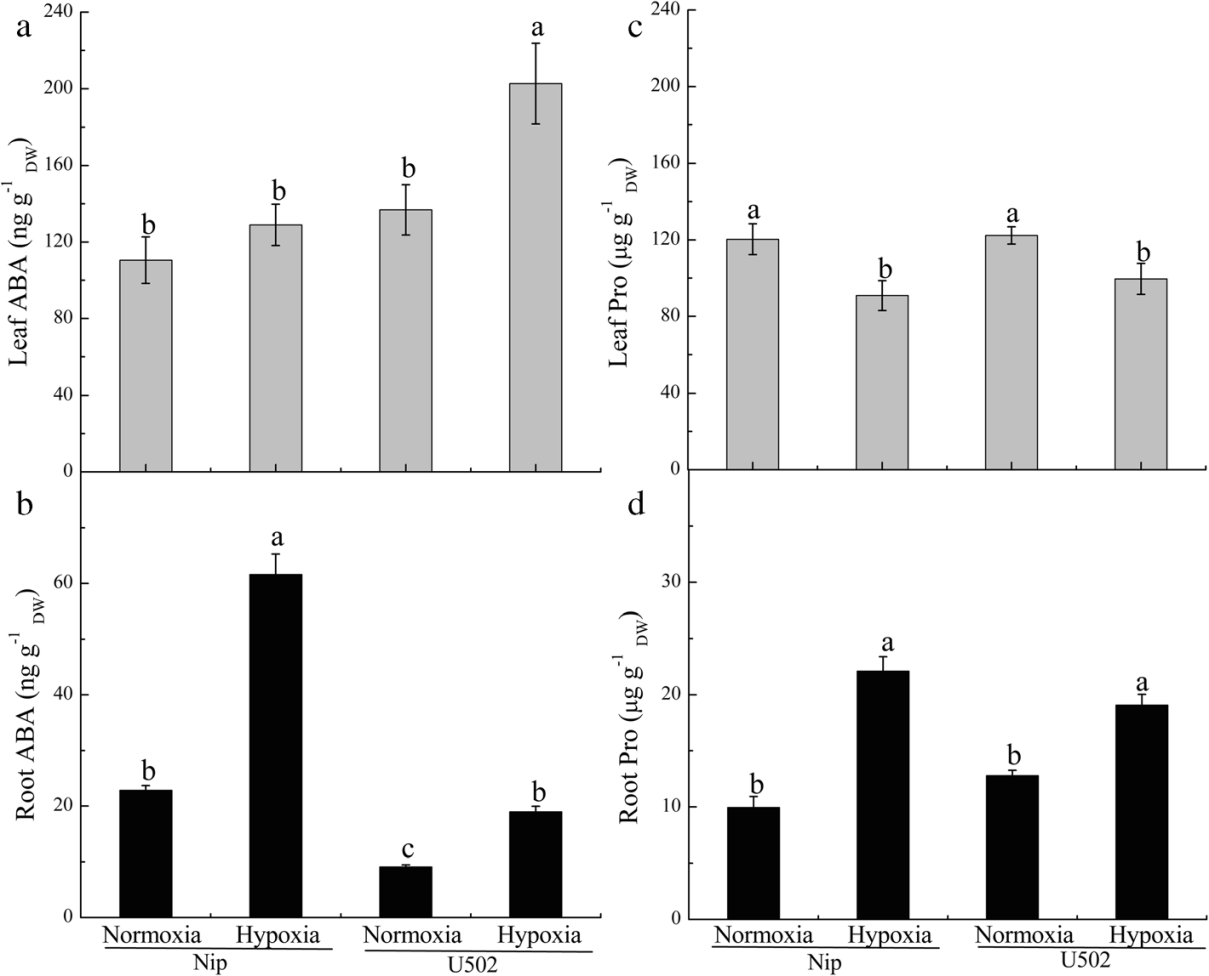
Effects of hypoxia on ABA and proline contents in the leaves (**a**, **c**) and roots (**b**, **d**) of both rice cultivars. DW represents dry weight. Data are the mean ± SE of three independent experiments. Different letters indicate significant differences at *P* < 0.05 using the least significant difference (LSD) test

**Fig. 3:**
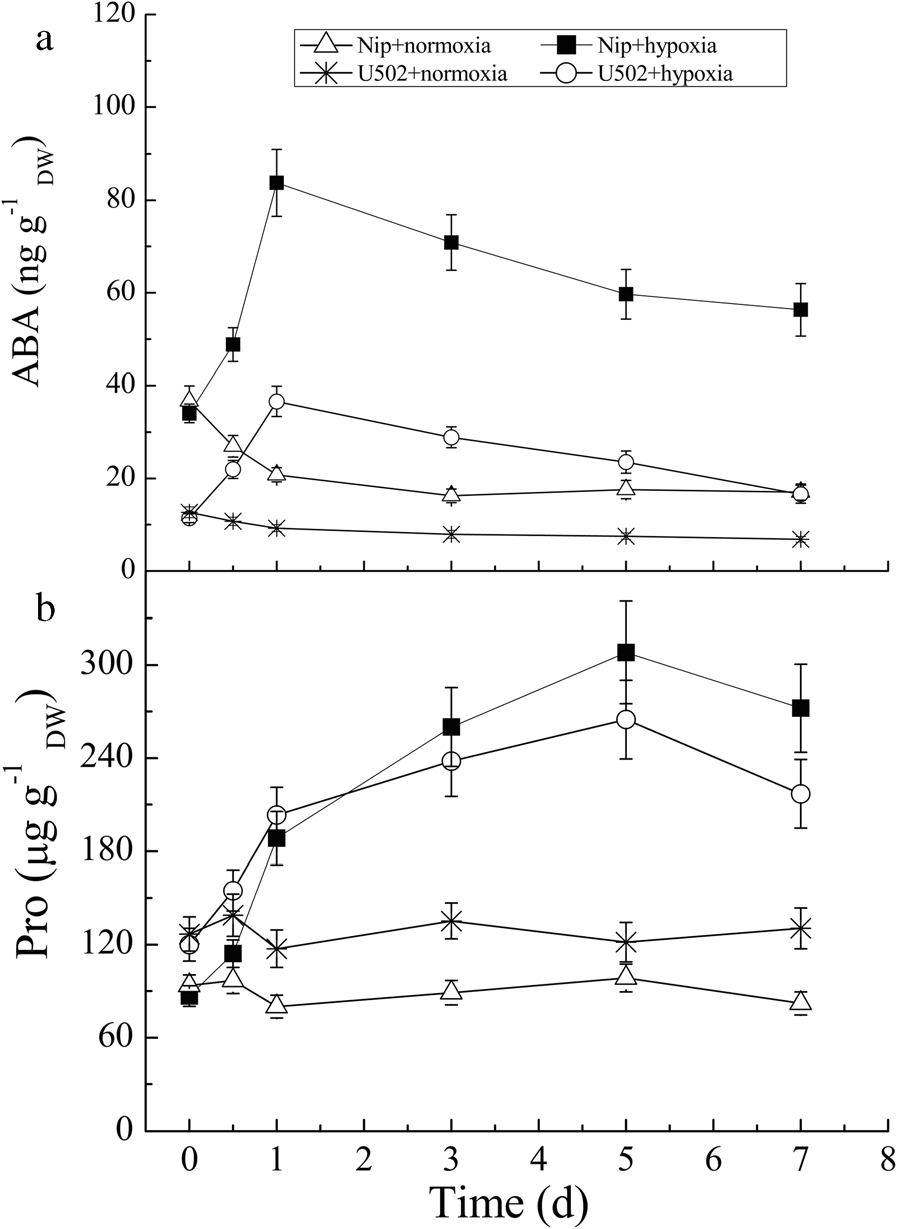
Time course of changes in ABA (**a**) and proline (**b**) contents in the roots of both cultivars subjected to normoxic and hypoxic conditions. DW represents dry weight. Data are the mean ± SE of three independent experiments

To assess the mechanism of tolerance to hypoxic stress, endogenous ABA and proline accumulation in the roots of both cultivars were monitored. Hypoxia induced significant accumulation of ABA and proline in the roots of both cultivars (Fig. 2b, d). Compared with the U502 cultivar, root ABA in Nip increased 2.25-fold after 14 d hypoxic stress, but their proline contents showed no significant differences. However, the stressed Nip cultivar maintained higher levels of proline synthesis than did the U502 cultivar in 3 d and 7 d of stress in experiment 2, as described in Fig. 3b. Hypoxia significantly inhibited the accumulation of proline in the leaves of both cultivars but stimulated the accumulation of ABA in the leaves of U502 (Fig. 2a, c). Analysis of gene expression demonstrated that hypoxia upregulated the expression of the genes *OsNCED3*, *OsNCED4* and *OsNCED5* in the Nip and of the *OsNCED4* and *OsNCED5* in the U502 (Fig. S1). These results were also in line with the variations in ABA production in the roots.

### Effects of exogenous ABA and ABA inhibitor on root proline production

To investigate the role of endogenous ABA and proline accumulation in the rice response to hypoxic stress, the time courses of ABA and proline accumulation were measured in both cultivars. Hypoxic stress simultaneously induced ABA and proline accumulation, but the time course variation trends of both molecules varied widely (Fig. 3). The root ABA contents rapidly increased and peaked after 1 d of hypoxic stress, after which the content gradually decreased over the next 6 d, and the levels in Nip were 1.29- and 2.40-fold higher than that in U502 after 1 d and 7 d of stress, respectively (Fig. 3a). The root proline contents also greatly increased under hypoxic stress, and peaked at 5 d of stress, but their contents varied little between the two genotypes except at 0.5 d and 1 d of stress (Fig. 3b). Under normoxic conditions, root ABA contents decreased slightly within the first 24 h but then remained constant, whereas the root proline contents varied little.

**Fig. 4:**
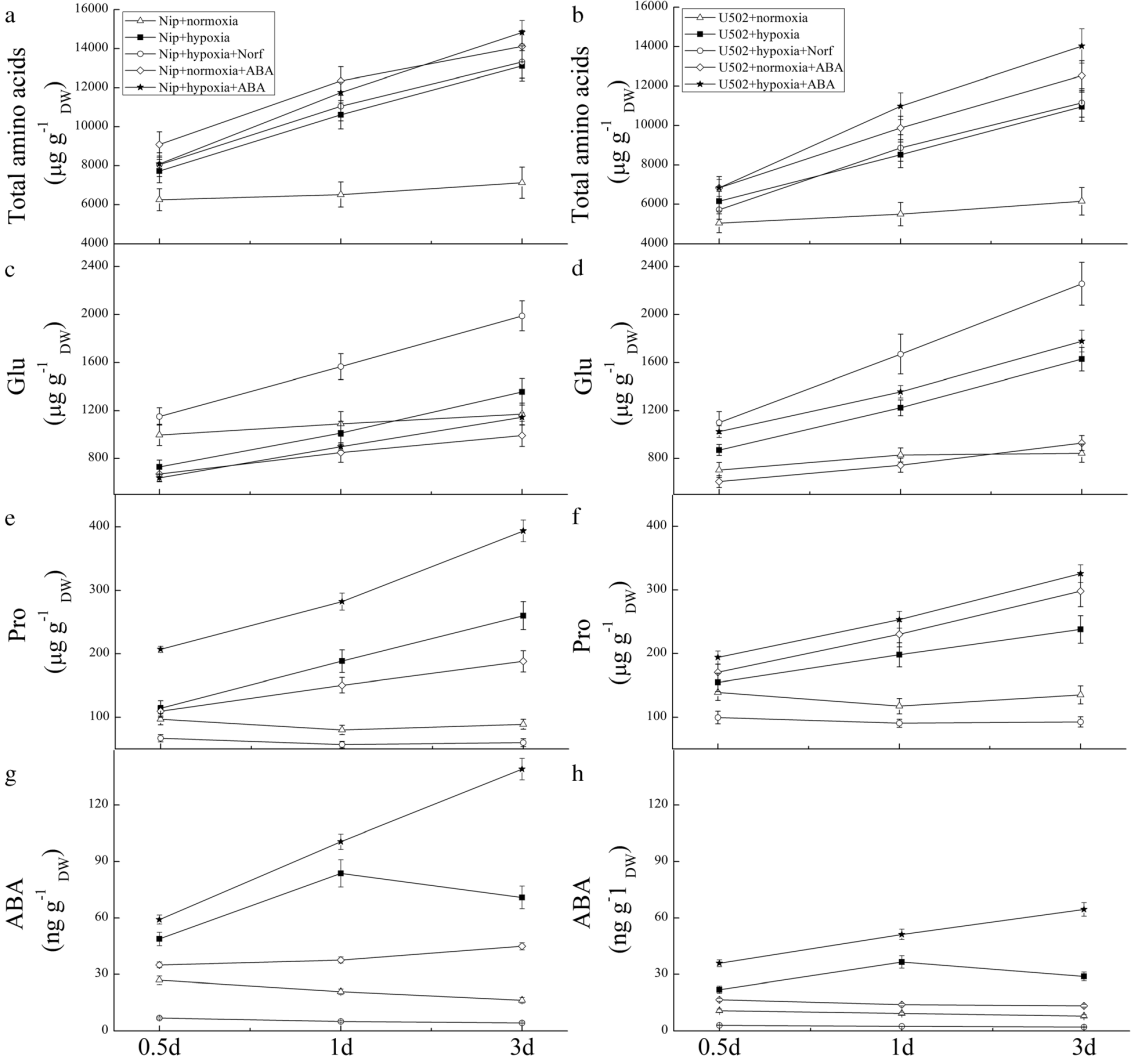
Effects of exogenous ABA and an ABA inhibitor on the content of total free amino acids, glutamate, proline and ABA in the roots of rice seedlings subjected to normoxic and hypoxic conditions. Amino acid (**a**, **b**), glutamate (**c**, **d**), proline (**e**, **f**) and ABA (**g**, **h**) contents in the roots of seedlings in the normoxia, hypoxia, hypoxia+norflurazon (Norf, 100 μM as ABA inhibitor), normoxia+ABA (ABA, 50 μM as ABA donor), and hypoxia+ABA (ABA, 50 μM as ABA donor) treatments. Measurements were performed at the indicated time points. DW represents dry weight. Data are the mean ± SE of three independent experiments

The variations in ABA and proline accumulation in roots over time raise the question of whether the increased accumulation of ABA acts as a signal to increase the production of proline under hypoxic stress. Therefore, the changes in the levels of total amino acids, glutamate, proline and ABA in response to the application of exogenous ABA or an ABA inhibitor (Norf) were quantified in a time course manner in experiment 2. As shown in Fig. 4, hypoxic stress induced a great increase in the total amino acid and proline content in both cultivars, with both showing higher levels in Nip than in U502 (Fig. 4). Under normoxic conditions, proline and glutamate production varied little with time, despite a slight increase in total amino acids. Hypoxia plus Norf treatment greatly inhibited the accumulation of proline in roots of both cultivars, whereas the glutamate (the main precursor of proline) content greatly increased. In contrast, treatment with exogenous ABA under hypoxia induced a great increase in endogenous proline and ABA content in comparison to the normoxia treatment, and the levels in the Nip were 1.21- and 2.16-fold higher than those in the U502 cultivar (Fig. 4c-h). Together, these results indicate that ABA may act upstream of proline during hypoxic stress.

### Effects of exogenous ABA and ABA inhibitor on the expression of genes encoding enzymes involved in proline biosynthesis and oxidative damage in roots

**Fig. 5:**
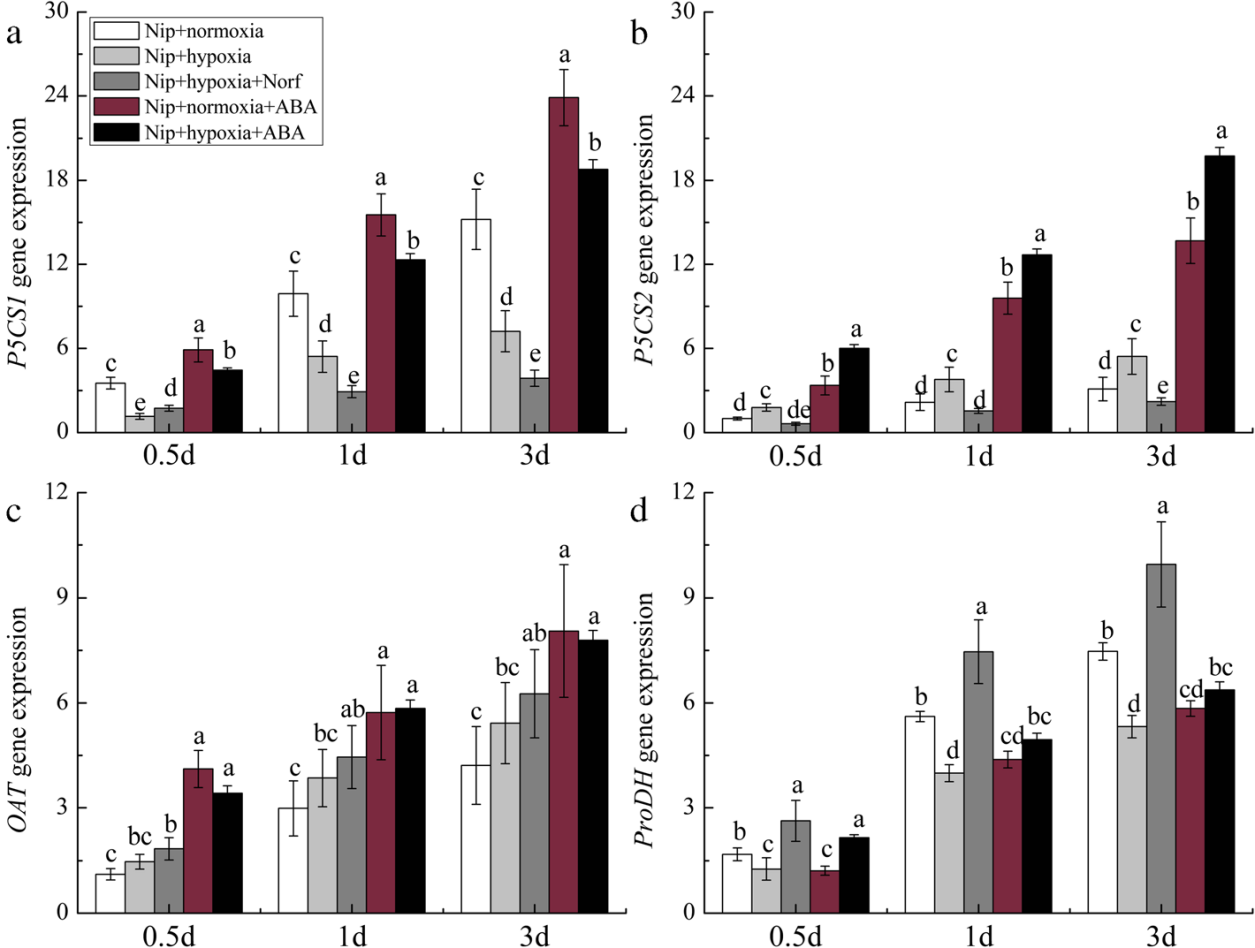
Effects of exogenous ABA and ABA inhibitor on the relative expression of genes related to proline metabolism in the Nip cultivar. *P5CS1* (**a**), *P5CS2* (**b**), *OAT* (**c**), and *ProDH* (**d**). DW represents dry weight. Data are the mean ± SE of three independent experiments. Different letters indicate significant differences at *P* < 0.05 using the least significant difference (LSD) test

**Fig. 6:**
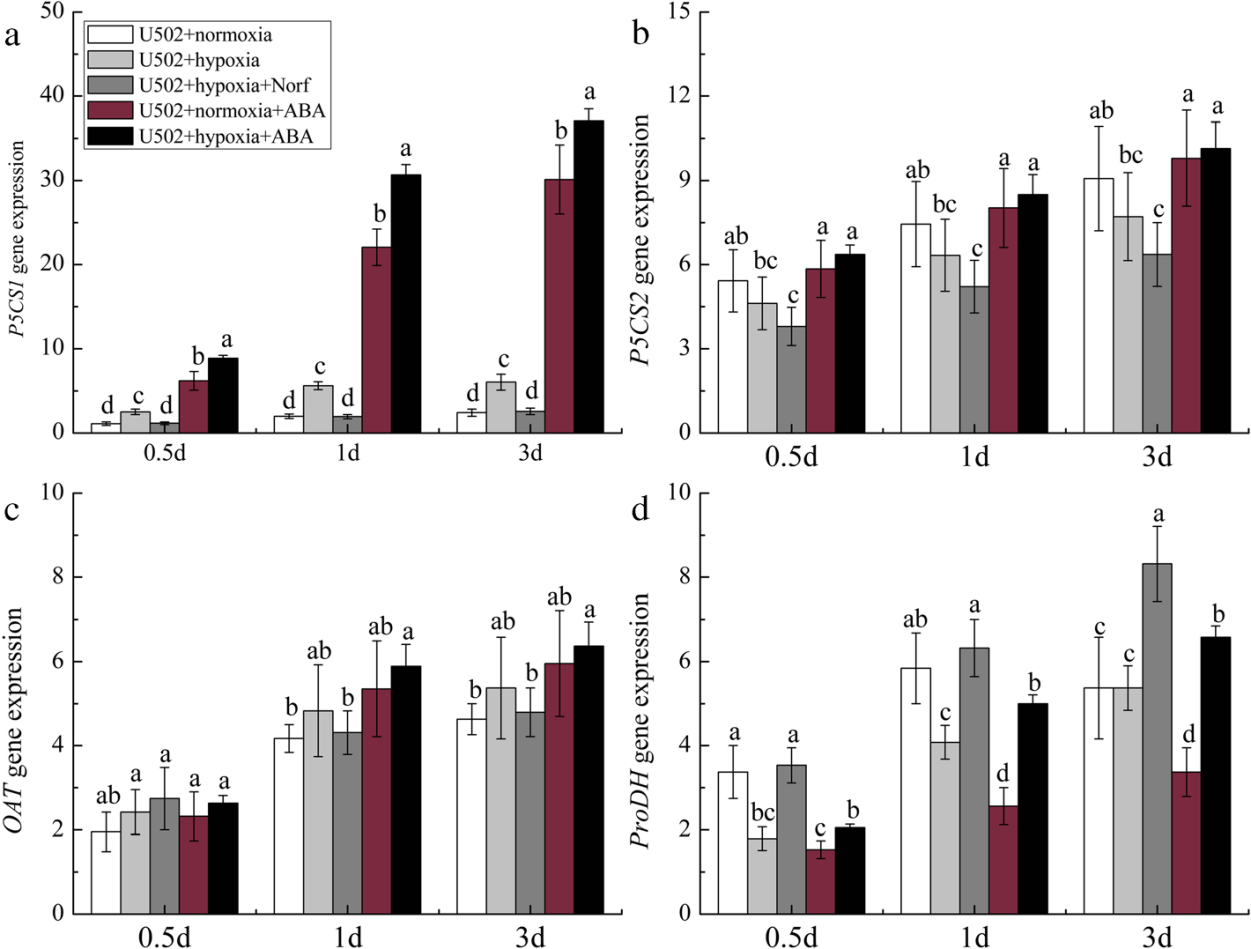
Effects of exogenous ABA and ABA inhibitor on the relative expression of genes related to proline metabolism in the U502 cultivar. *P5CS1* (**a**), *P5CS2* (**b**), *OAT* (**c**), and *ProDH* (**d**). DW represents dry weight. Data are the mean ± SE of three independent experiments. Different letters indicate significant differences at *P* < 0.05 using the least significant difference (LSD) test

Compared with the normoxia treatment, hypoxia treatment induced a significant increase in P5CS activity and a decrease in ProDH activity in the roots of both cultivars (Fig. S2), resulting in increased proline contents. Further results showed that hypoxic stress downregulated the expression of *OsP5CS1* and *OsProDH* but upregulated that of *OsP5CS2* in Nip (Fig. 5a, b, d). Hypoxia plus Norf treatment significantly downregulated the expression of *OsP5CS1* and *OsP5CS2* but upregulated that of *OsProDH*, correspondingly inhibiting the production of endogenous proline, whereas exogenous ABA significantly upregulated the expression of *OsP5CS1*, *OsP5CS2*, and *OsProDH*. In the U502 cultivar, hypoxia significantly upregulated the expression of *OsP5CS1* but downregulated that of *OsProDH* (Fig. 6a, d) compared with the normoxia treatment. Hypoxia plus Norf treatment downregulated the expression of *OsP5CS1* but upregulated that of *OsProDH*, but these effects were reversed by exogenous ABA plus hypoxia treatment. All of the results indicate that the significant differences in gene expression associated with proline metabolism are mediated by ABA signaling. In Nip, hypoxia-induced proline accumulation in roots was attributed to the upregulation of Os*P5CS2* and downregulation of Os*ProDH*, whereas upregulation of Os*P5CS1* combined with downregulation of Os*ProDH* enhanced the proline level in U502.

**Fig. 7:**
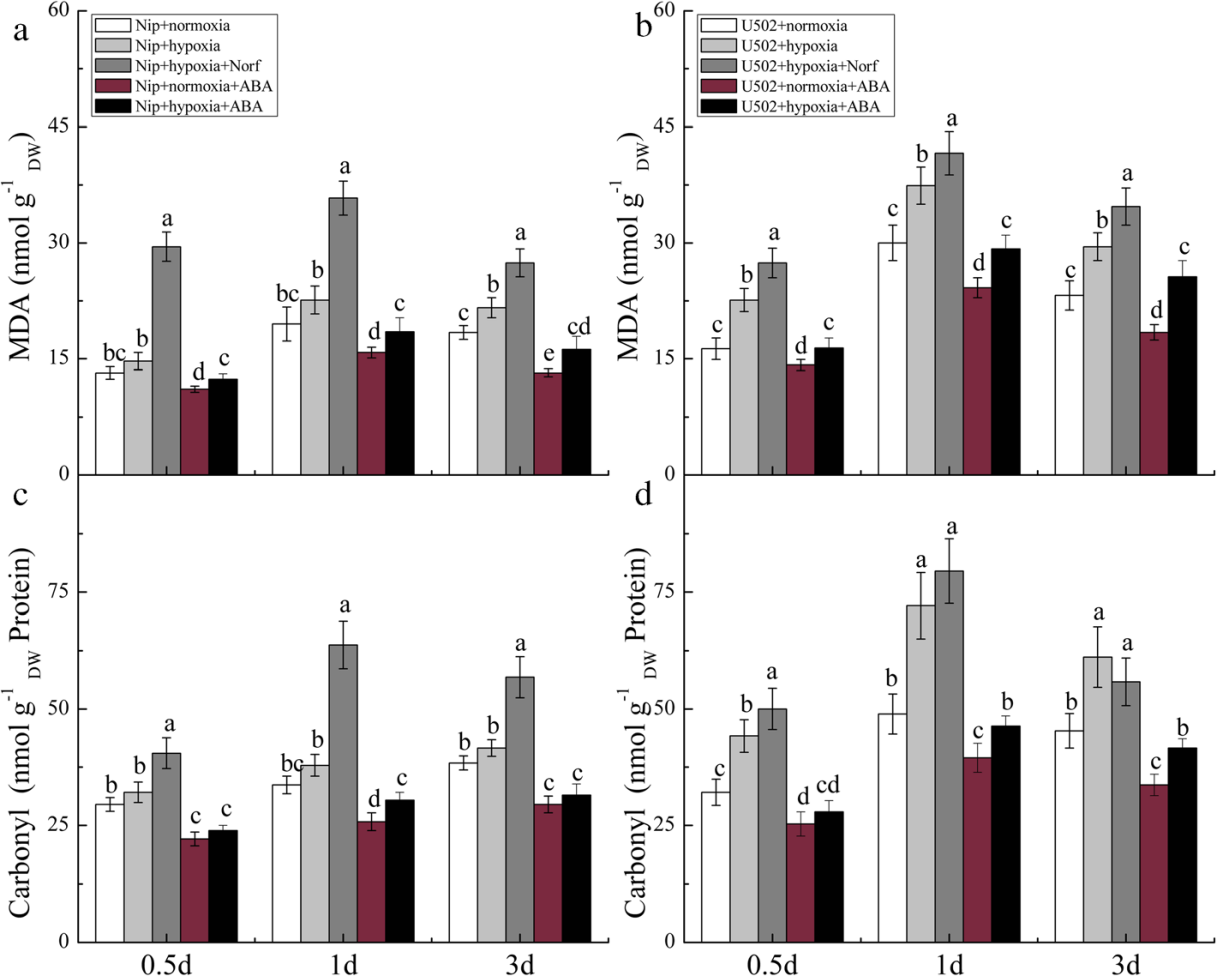
Effects of exogenous ABA and ABA inhibitor on oxidative damage in both cultivars. The contents of MDA (**a**, **b**) and carbonyl groups (**c**, **d**) represent the lipid peroxidation and protein oxidation, respectively. DW represents dry weight. Data are the mean ± SE of three independent experiments. Different letters indicate significant differences at *P* < 0.05 using the least significant difference (LSD) test

To further investigate the effects of exogenous ABA and ABA inhibitor on rice tolerance to hypoxia, we measured lipid peroxidation and protein oxidation in response to the different treatments. After 0.5–3 d of hypoxic stress, the variations in root MDA and carbonyl group contents were consistent with the trends observed in experiment 1 (Fig. 7a-d). Hypoxia plus Norf treatment significantly aggravated the oxidative damage in both cultivars in comparison to that in hypoxia treatment. However, exogenous ABA significantly alleviated the root oxidative damage, which is in line with the observed variations in ABA and proline accumulation. The results further confirm that ABA participates and plays a crucial role in reducing oxidative damage under hypoxic stress.

## Discussion

### The Nip cultivar seems more tolerant to hypoxia than the U502 cultivar, with higher ABA accumulation and lower oxidative damage in roots

Under hypoxic stress, symptoms such as chlorosis, arrested nutrient uptake and reduced growth occur in plants because aerobic respiration and mitosis in all eukaryotic cells are significantly inhibited due to O_2_ deficiency [9]. Hypoxic stress also induced significantly reduced leaf photosynthesis and root activity in both cultivars, which is in agreement with previous findings obtained under flooding conditions [36, 37]. However, the response of rice growth to hypoxic stress showed significant genotypic differences, and the Nip cultivar is seemingly more tolerant to hypoxic stress than the U502 cultivar. The relative higher rice biomass of the Nip cultivar was accompanied by adaptive traits, such as adventitious root development [12] and increased photosynthesis [8]. High photosynthetic rates enhance the long-distance translocation of primary photoassimilates from the phloem to the roots [38]. In contrast, the higher ABA content in leaves of the U502 cultivar leads to stomatal closure, inhibiting the photosynthetic capacity and carbohydrate production [8]. This retardation may also result from the relatively high cellular oxidative damage in the U502 cultivar, whereby MDA and carbonyl groups reflect lipid peroxidation and protein oxidation, respectively (Table 1). However, the higher oxidative damage in the U502 cultivar than in the Nip cultivar was accompanied by higher activities of the SOD and CAT enzymes that serve as significant antioxidant enzymes, which is contrary to the previous results [6, 39]. The results suggested that the accumulation of these antioxidant enzymes is still not sufficient for scavenging the ROS accumulated under hypoxia, which correspondingly stimulates their increased accumulation.

Under abiotic stress, a temporary increase in the ABA and proline content also plays an important role in enhancing plant adaptation to hypoxia tolerance [40, 41]. In our study, both cultivars displayed significantly enhanced accumulation of proline and ABA in roots, indicating a stimulatory effect of hypoxia on their accumulation. The expression of *NCED* (e.g., *OsNCED3*, *OsNCED4* and *OsNCED5*) genes, which control the first step of the ABA biosynthesis pathway, also supports the conclusion that ABA synthesis is activated under hypoxic stress. Regardless of the presence of hypoxic stress, the ABA contents showed significant genotypic differences, and the value in Nip roots was significantly greater than that in U502 roots. This result was consistent with a previous result [33], which attributed the low levels of root-sourced ABA in upland rice to the high level of root exudation, thus improving ABA-dependent drought adaptation. The response of ABA and proline to hypoxic stress in roots suggests that rice growth is tightly regulated by internal signals, with ABA likely playing an essential role in rice acclimation to hypoxic stress. However, the role of proline and ABA and their relationship in rice acclimation to hypoxic stress remain unclear.

### Root ABA acts upstream of proline during hypoxic stress

Using classical toxicology, our study further demonstrates that root ABA may act upstream of proline metabolism during hypoxic stress. First, the content of endogenous proline and ABA increased simultaneously in a time course manner under hypoxic stress. Within 24 h of hypoxic stress, the root ABA and proline content greatly increased. Other reports have described a similar increase in plant ABA content and hypoxic tolerance under hypoxic stress conditions [27, 42]. However, they demonstrated that the ABA-induced anaerobic tolerance was inhibited by the exogenous application of cycloheximide, which further indicates that ABA likely serve as an early signal substance to sensing hypoxic stress and thus inducing physiological adaptation. In our study, peak ABA production occurred before proline was accumulated in both cultivars (Fig. 3). This finding indicates that cross talk between the ABA and proline signaling pathways is likely involved in hypoxia-induced acclimation during rice growth. Although ABA accumulation after 7 d of hypoxic stress in Nip was significantly higher than that in U502, their variation in response to exogenous Norf or ABA treatment presented similar trends. Compared with the hypoxia treatment, the accumulation of proline increased significantly under exogenous ABA treatment but decreased notably under Norf treatment. Given the role of proline as an osmolyte and its ability to balance intracellular redox homeostasis under different stress conditions [25, 43], our results indicate that ABA could initiate a network of signaling pathways involved in proline metabolism, thus regulating rice acclimation to hypoxic stress. Similar relationships between ABA and proline and their involvement in abiotic adjustment have also been observed in other crop plants [30, 44, 45].

Hypoxic roots also undergo several biochemical modifications related to N uptake and assimilation, which correspondingly participate in plant acclimation to hypoxic stress [7, 46]. In our study, hypoxia induced a significant increase in the contents of total amino acids in roots. Such stress-induced amino acid accumulation might be a mechanism to provide cells with precursors for several compounds known to be involved in abiotic stress responses [46, 47], such as polyamines or metabolites of secondary metabolism. The content of glutamate, the main precursor of proline, increased by 70.0% in Nip and 167.2% in U502 after 3 d of hypoxic stress in comparison to normoxic conditions, thus facilitating nitrogen recycling [24, 48]. Hypoxia plus Norf treatment induced a significant increase in glutamate over time but conversely inhibited proline synthesis in both cultivars. However, exogenous ABA treatment greatly suppressed the hypoxia-induced accumulation of glutamate in roots, especially at 1 and 3 d of stress. The results indicate that Norf-induced glutamate accumulation appears to depend on the inhibition of ABA biosynthesis, which also indirectly confirms the aforementioned conclusion that proline likely acts downstream of ABA. In addition, the accumulation of amino acids mediated by ABA in the Nip cultivar was much greater than that in the U502 cultivar, also playing an important role in alleviating oxidative damage under hypoxic stress [23, 46].

### ABA alleviates hypoxia-induced oxidative damage and mediates the expression of the genes involved in proline metabolism

In general, proline accumulation results from the enhanced activity of P5CS, the rate-limiting enzyme involved in proline biosynthesis, or a decrease in ProDH activity [21, 25]. Our study demonstrated that the elevated P5CS and suppressed ProDH activities under hypoxic stress jointly induced the accumulation of proline in the roots of both cultivars. Using transgenic technology, Aleksza et al. [49] reported that the proline content was reduced in the *P5CS1–1* mutant and enhanced in the *PDH2–2* mutant, suggesting that these two genes determine proline synthesis under abiotic stress. To determine whether ABA indeed correlates with coordinated inhibition of *ProDH* and induction of *P5CS* gene expression, the signaling cues that regulate this process were also investigated. The expression of *P5CS2* in the Nip cultivar and *P5CS1* in the U502 cultivar was significantly upregulated under hypoxic stress, which was further strengthened by the application of exogenous ABA but was reversed by blocking ABA biosynthesis in the hypoxia plus Norf treatment. However, *ProDH* expression showed the opposite trends in the aforementioned different scenarios. Previous studies of *P5CS* genes revealed significant differences in the temporal and spatial regulation of their transcription [30, 50], our study found that the expression of the *P5CS1* and P5CS2 genes showed the significant genotypic differences. Székely et al. contributed the difference to the distinct cell-type-specific and subcellular localization patterns in root tips, leaves and flower organs of Arabidopsis [21]. In addition, the transcription of P5CS genes is differentially regulated by drought, salinity and ABA, suggesting that these genes play specific roles in the control of proline biosynthesis [21]. Although the intrinsic mechanisms for the differences in gene expression still require elaboration, our results suggest that proline accumulation and the expression of the genes encoding enzymes of the proline biosynthesis pathway, particularly the Os*P5CS1*, Os*P5CS2* and Os*ProDH*, require ABA for induction under hypoxic stress, which is consistent with the results recently obtained by other authors [24, 51].

Under different abiotic stress conditions, elevated proline metabolism, antioxidative enzyme activities, and gene expression levels in response to abiotic stress have been widely reported in plants [16, 43, 47]. Our results further demonstrate that ABA accumulation induced by hypoxia was able to enhance rice tolerance to hypoxia by reducing oxidative damage in roots, especially in the Nip cultivar. Under hypoxic conditions, root oxidative damage was significantly enhanced in the U502 cultivar but not in the Nip cultivar in comparison to that under normoxic conditions. However, hypoxia plus Norf treatment significantly aggravated root oxidative damage by enhancing the content of MDA and carbonyl groups in both cultivars. These findings indicate that ABA may play an important role in alleviating hypoxia-induced oxidative damage in roots, which was further confirmed by subsequent experiments in which the application of exogenous ABA significantly alleviated hypoxia-induced oxidative damage.

### Potential mechanisms of adaptation to hypoxic stress in the Nip and U502 cultivars

The Nip cultivar is more tolerant to hypoxia than U502, as evidenced by the high dry matter content and lower root oxidative damage. Focusing on the root response to ABA and proline, our results demonstrated that, regardless of the differences in rice biomass and proline level, root ABA could act upstream of proline accumulation by regulating the expression of genes involved in proline metabolism and significantly alleviate hypoxia-induced oxidative damage in both cultivars. The root ABA level in Nip was significantly higher than that in U502, which is consistent with the variation in rice biomass. However, the link between growth and ABA content in aerial parts was not as obviously consistent as it was in roots. Hypoxia significantly enhanced the ABA content in the leaves of the U502 cultivar but not in the Nip cultivar. Das and Kar [52] revealed that by affecting NADPH oxidase generated apoplastic ROS, ABA mediates differential growth responses in roots and shoots of Wilczek seedlings under water stress. The hypoxia-induced decline of photosynthesis in U502 was probably associated with higher ABA content that lead to stomatal closure [8], which further reduces the production and translocation of primary photoassimilates in rice leaves. Therefore, we conclude that the more tolerant Nip cultivar utilizes a better protective mechanism than the hypoxia-sensitive U502 cultivar for retaining higher photosynthetic and antioxidant capacity mediated by endogenous ABA.

Although many physiological studies have suggested that proline is involved in multiple stress protection mechanisms, the values showed no significant difference between the two rice cultivars in our study. In previous reports, higher accumulation of proline was found to be correlated with improved stress tolerance [53], whereas in others, such a correlation was not apparent [54]. These interesting findings suggest that proline accumulation may not be the sole factor for adaptation to environmental stress. Under hypoxic conditions, perhaps other signaling pathways in roots are also activated to reduce the negative effects of hypoxia. The different protective mechanisms controlled by ethylene, IAA and nitric oxide (NO) have been widely demonstrated in plant adaptations to abiotic stresses [55–57]. Some authors demonstrated that NO or ethylene likely acts as a downstream ABA signal molecule and participates in signal transduction processes, thus increasing plant antioxidant ability [10, 58]. Therefore, whether hypoxia or ABA further stimulates the NO/ethylene production and their roles in enhancing Nip cultivar tolerance to hypoxia still need to be elucidated.

## Conclusions

In conclusion, our plant growth data clearly show that the Nip cultivar grew better with higher biomass and leaf photosynthesis and was more adaptive to hypoxic stress than the U502 cultivar, which was related to the higher ABA amounts and enhanced ABA-mediated antioxidant capacity in roots of the former cultivar. The results also demonstrate that root ABA could act upstream of proline accumulation by regulating the expression of the genes involved in proline metabolism, which likely improves rice acclimation to hypoxic stress to a certain extent, especially in the Nip cultivar. However, the proline level in Nip showed no significant difference from that in U502, indicating that proline accumulation may not be the sole factor for adaptation to hypoxic stress. Therefore, other signaling pathways that enhance the tolerance of the Nip cultivar to hypoxia still need to be elucidated.

## Methods

### Plant materials and growth conditions

The traditional lowland japonica rice cultivar Nipponbare (Nip) and upland japonica rice cultivar upland 502 (U502) were used in this study. Seeds of the Nip and U502 cultivars were obtained from China National Center for Rice Improvement (http://www.chinariceinfo.com/en/AboutUs/Organization/8036.html). Rice seeds were surface sterilized with a 1% (v/v) aqueous sodium hypochlorite solution. Germinated seeds were transferred to a solution of 0.5 mM CaCl_2_ (pH 5.5) for well growth of rice roots. After 3 d, seedlings were transplanted into 1-L black plastic pots that contained a solution composed of NH_4_NO_3_ (0.5 mM), NaH_2_PO_4_·2H_2_O (0.18 mM), KCl (0.18 mM), CaCl_2_ (0.36 mM), MgSO_4_·7H_2_O (0.6 mM), MnCl_2_·4H_2_O (9 μM), Na_2_MoO_4_·4H_2_O (0.1 μM), H_3_BO_3_ (10 μM), ZnSO_4_·7H_2_O (0.7 μM), CuSO_4_ (0.3 μM), and FeSO_4_·7H_2_O-ethylenediaminetetraacetic acid (EDTA) (20 μM) [55]. There were 5 seedlings per pot. All seedlings were cultivated in a growth chamber under the following conditions: 14-h/10-h light/dark photoperiod, 400 μmol m^− 2^ s^− 1^ light intensity, 28 °C daytime and 23 °C nighttime temperature, and 60% relative humidity (RH). The solution pH was adjusted to 5.5 with 5 mM 2-(N-morpholino) ethanesulfonic acid (MES).

**Experiment 1:** After 1 week of cultivation, six pots with similarly sized seedlings were selected for the experiment 1, and the six replications of each genotype were equally divided into two groups: normoxia and hypoxia treatments. For the normoxia treatment, three of the pots containing each genotype were aerated with an air pump, and the solution was aerated every 4 h to maintain the dissolved O_2_ content within a range of 1.5–2.0 mg L^− 1^. For the hypoxia treatment, N_2_ gas was pumped into the three pots every 4 h, and the dissolved O_2_ content was maintained at 0.1 ~ 0.5 mg L^− 1^. The solutions were replaced every 3 d. The dissolved O_2_ content was measured using a portable dissolved oxygen meter (HI9143; Hanna Instruments, Padova, Italy).

After 14 d of cultivation under normoxia and hypoxia, leaf gas exchange was measured on the youngest fully expanded leaf using a Li-6400XT portable photosynthesis system (Li-Cor Co. Ltd. UAS). Measurements were performed from 09:00 to 12:00 h with a photosynthetic photon flux density of 1500 mmol m^− 2^ s^− 1^, cuvette temperature of 28 °C, reference CO_2_ concentration of 390 mmol mol^− 1^, and the relative humidity of 60–70%. Leaf SPAD was measured with a chlorophyll meter (SPAD502 Plus; Spectrum Technologies Inc., Aurora, IL, USA). After determination of leaf photosynthesis and SPAD, rice shoots (leaf blades and leaf sheaths) and roots were harvested separately, frozen immediately in liquid nitrogen, and stored at − 70 °C until use.

**Experiment 2:** To measure the time course of production of ABA and proline in roots, after 1 week of cultivation, similar pretreated rice seedlings of each genotype were grown under hypoxic and normoxic conditions, as described in experiment 1. The roots of these seedlings were sampled at 0, 0.5, 1, 3, 5, and 7 d, respectively, and the contents of ABA and proline was determined.

To investigate the cross-talk between ABA and proline in rice acclimation to hypoxic stress, after 1 week of precultivation, similar pretreated rice seedlings of each genotype were also subjected to the following five treatments: normoxia, hypoxia, hypoxia+Norf (100 μM Norf, as an ABA synthesis inhibitor), normoxia+ABA (50 μM ABA, as an ABA donor), and hypoxia+ABA (50 μM ABA, as an ABA donor) [59]. Because the production of ABA peaked at 24 h after hypoxic stress (see the Results section), the roots of seedlings in the five treatments were sampled after 0.5, 1 and 3 d of cultivation. Then, the total amino acids, proline, glutamat, ABA, proline-related metabolic enzymes, gene expression and root oxidative damage (malondialdehyde (MDA) and carbonyl groups) levels were measured.

### Root activity, oxidative damage and the activities of antioxidative enzymes

Root activity was determined using the triphenyl tetrazolium chloride (TTC) method, as described by Wang et al. [60]. In brief, 0.5 g fresh root sample was immersed in 10 mL of an equally mixed solution of 0.4% TTC and phosphate buffer and kept in the dark at 37 °C for 2 h. The reaction was stopped with 2 mL of 1 mol L^− 1^ H_2_SO_4_. Roots were dried with filter paper, and ethyl acetate extraction was performed. The absorbance of the extract at 485 nm was recorded. Root activity was calculated as TTC reduction intensity, and the result is expressed as the amount of TTC reduction (μg) per dry root weight (g) and time (h).

In accordance with the method of Velikova et al. [61], lipid peroxidation was determined via measurement of MDA content resulting from reactions involving thiobarbituric acid (TBA). Oxidative damage to proteins was estimated based on the content of carbonyl groups, as described by Zhang et al. [62]. The protein content was determined according to the method of Bradford [63], with bovine serum albumin used as the standard.

Samples of fresh roots (0.5 g) was homogenized with 5 mL 10 mM phosphate buffer (pH 7.0) containing 4% (w/v) polyvinylpyrrolidone and 1 mM EDTA. The homogenate was centrifuged at 12, 000 rpm for 15 min at 4 °C, then stored at − 70 °C for the determination of the activities of antioxidant enzymes. The activities of SOD, CAT, APX, and POD were estimated using the photocolorimetric method of Jiang and Zhang [64]. All of these measurements were performed in three independent biological replicates.

### Amino acids and ABA analysis

The contents of proline, glutamate and total free amino acid in roots were determined as follows: approximately 1.0 g of fresh roots was powered in liquid N_2_, homogenized with 3% sulfosalicylic acid (w/v) for 12 h at 4 °C. The homogenate was centrifuged at 10,000 rpm for 10 min at 4 °C, then passed through a 0.22-μm aqueous film filter. Amino acid content was determined using a Hitachi L-8900 automatic amino acid analyzer (L-8900; Hitachi Corp., Tokyo, Japan), according to the method described in Ma et al. [65].

ABA contents in roots and leaves were quantified using a high-performance liquid chromatography-tandem mass spectrometry system (HPLC-MS). ABA extraction, purification, and determination were performed according to Cao et al. [66]. Each treatment had three replications.

### P5CS, OAT and ProDH activities

Approximately 0.5 g of fresh root sample with three replications was extracted with 5 mL of extraction buffer comprising 50 mM Tris-HCl (pH 7.4), 7 mM MgCl_2_, 0.6 M KCl, 3 mM EDTA, 1 mM dithiothreitol and 5% (w/v) in soluble polyvinylpolypyrrolidone. The homogenates were centrifuged at 39,000 rpm for 5 min, after which the supernatants were further clarified by centrifugation at 39,000 rpm for 20 min at 4 °C. The activities of 1-pyrroline-5-carboxylate synthase (P5CS, EC2.7.2.11), ornithine aminotransferase (OAT, EC2.6.1.68) and proline dehydrogenase (ProDH, EC1.5.99.8) in the supernatant were measured in accordance with a previously reported method [67].

### Quantitative real-time PCR

Total RNA extraction, reverse transcription, and PCR were performed according to Cao et al. [47]. Primers were designed to amplify 150- to 250-bp fragments using PRIMER5 software [68]. The primers used in the assays are listed in Supplementary Table S1. Expression levels were normalized to that of the reference gene UBQ using the primers UBQfw (5′-GCTCCGTGGCGGTATCAT-3′) and UBQrv (5′-CGGCAGTTGACAGCCCTAG-3′) [69]. The 2^−ΔΔCT^ method was employed to determine the relative gene transcript levels with the mean value of triplicate experiments.

### Statistical analysis

Data were analyzed by one-way ANOVA, and the mean values were compared by the least significant difference (LSD) test using SPSS v. 13.0 (IBM Corp., Armonk, NY, USA). Different letters on the figures indicate that the mean values were statistically different at the *P* < 0.05 level. The figures were drawn using Origin v. 8.0 (Origin Lab Corporation, Northampton, MA, USA).

## Supplementary information

**Additional file 1:Table S1.** RT-qPCR primers used in this study.

**Additional file 2:Figure S1.** Expression of the genes related to the ABA biosynthesis in roots.

**Additional file 3:Figure S2.** Activities of P5CS, OAT and ProDH enzymes involved in proline metabolism.

## Data Availability

The datasets used and/or analysed during the current study available from the corresponding author on reasonable request.

## Abbreviations

ABA: Abscisic acid
U502: Upland 502
Nip: Nipponbare
Norf: Norflurazon
O_2_: Oxygen
ROS: Reactive oxygen species
CAT: Hydrogen peroxidase
APX: Ascorbate peroxidase
POD: Peroxidase
SOD: Superoxide dismutase
MDA: Lipid peroxidation
P5CS: 1-pyrroline-5-carboxylate synthase
OAT: Ornithine aminotransferase
ProDH: Proline dehydrogenase
EDTA: Ethylenediaminetetraacetic acid
MES: 2-(N-morpholino) ethanesulfonic acid
RH: Relative humidity
MDA: Malondialdehyde
TTC: Triphenyl tetrazolium chloride
TBA: Thiobarbituric acid
HPLC: High-performance liquid chromatography
DW: Dry weight
