# Supplementary material for: Abscisic acid mediated proline biosynthesis and antioxidant ability in roots of two different rice genotypes under hypoxic stress
Source: BMC Plant Biol. 2020 May 8;20:198. doi: 10.1186/s12870-020-02414-3 (PMC7206686; doi:10.1186/s12870-020-02414-3)
Supplement: Supplementary file 1 — Additional file 1:Table S1. RT-qPCR primers used in this study. [file 12870_2020_2414_MOESM1_ESM.docx]

Table S1. Gene-specific primers used for the real-time polymerase chain reaction analysis

| Gene name |  | Primer sequence (5’-3’) |
| --- | --- | --- |
| *OsP5CS1* | Forward  Reverse | AAGAGGTTGATGTACGCGAGATG |
|  |  | CCGAAGACACGGCCTGAA |
| *OsP5CS2* | Forward  Reverse | GAGAGGGAACGGCCAAGTG |
|  |  | CAGATCCTTGTGTGTATA |
| *OsOAT* | Forward  Reverse | GCCAAGTCAACACATGACACAA |
|  |  | TGTTGGATCTCATCCGCACTTA |
| *OsProDH* | Forward  Reverse | CCAACGTCCACGCTGATAAGA |
|  |  | ACAGGTCCTATAGCCGTTGCA |
| *OsNCED1* | Forward  Reverse | CTCACCATGAAGTCCATGAGGCTT |
|  |  | GTTCTCGTAGTCTTGGTCTTGGCT |
| *OsNCED2* | Forward  Reverse | GGTATGGAAACGAGGATAGTGGTT |
|  |  | TGCTTATTGTTGTGCGAGAAGTTC |
| *OsNCED3* | Forward  Reverse | CCCCTCCCAAACCATCCAAACCGA |
|  |  | TGTGAGCATATCCTGGCGTCGTGA |
| *OsNCED4* | Forward  Reverse | TCCATCTCCTTCTCCCTCCTCCCA |
|  |  | CCTCGCACCCTGCTTGATCTTGCC |
| *OsNCED5* | Forward  Reverse | ACATCCGAGCTCCTCGTCGTGAA |
|  |  | TTGGAAGGTGTTTTGGAATGAACCA |
